# Association of Stress, Glucocorticoid Receptor, and FK506 Binding Protein Gene Polymorphisms With Internalizing Disorders Among HIV-Infected Children and Adolescents From Kampala and Masaka Districts—Uganda

**DOI:** 10.3389/fped.2021.666426

**Published:** 2021-10-26

**Authors:** Tonny Jimmy Owalla, Wilber Joseph Ssebajjwe, Dennis Muhanguzi, Jacqueline Samantha Womersley, Eugene Kinyanda, Allan Kalungi

**Affiliations:** ^1^Mental Health Unit, Medical Research Council/Uganda Virus Research Institute and London School of Hygiene and Tropical Medicine Uganda Research Unit, Entebbe, Uganda; ^2^Med Biotech Laboratories, Kampala, Uganda; ^3^Department of Bio-Molecular Resources and Bio-Laboratory Sciences, College of Veterinary Medicine, Animal Resources and Biosecurity, Makerere University, Kampala, Uganda; ^4^Department of Psychiatry, Faculty of Medicine and Health Sciences, Stellenbosch University, Cape Town, South Africa; ^5^South African Medical Research Council, Stellenbosch University Genomics of Brain Disorders Research Unit, Faculty of Medicine and Health Sciences, Stellenbosch University, Cape Town, South Africa; ^6^Department of Psychiatry, College of Health Sciences, Makerere University, Kampala, Uganda; ^7^Department of Immunology and Microbiology, College of Health Sciences, Makerere University, Kampala, Uganda

**Keywords:** stress, NR3C1, FKBP5, internalizing disorders, children, depression, anxiety

## Abstract

Children and adolescents living with human immunodeficiency virus (CA-HIV) suffer a considerable burden of internalizing disorders (IDs; depressive and anxiety disorders). Environmental and genetic factors have been reported to influence the vulnerability to IDs in western settings; however, their role among African populations remains inadequately explored. We investigated the individual and interactive effects of stress and single-nucleotide polymorphisms within the FK506 binding protein 5 (rs1360780) and glucocorticoid receptor (rs10482605) genes on ID status in a cohort of CA-HIV in Uganda. We genotyped rs10482605 (309 cases and 315 controls) and rs1360780 (350 cases and 335 controls) among CA-HIV with and without IDs using Kompetitive Allele-Specific PCR. Socio-demographic variables, as well as allele and genotype distributions, were compared between cases and controls using chi-square tests. Genotypes were assessed for Hardy–Weinberg equilibrium. Composite indices of recent and chronic stress classes were also generated. A hierarchical cluster analysis was used to generate cutoff points within each of the indices of recent and chronic stress. Logistic regression was used to assess the association between IDs and each of recent stress, chronic stress, and the investigated genotypes. The interaction effect of chronic/recent stress on the association between each of the polymorphisms and IDs was determined using a likelihood ratio test. We observed no significant association between IDs and rs1360780 and rs10482605 polymorphisms within the FKBP5 and glucocorticoid receptor genes, respectively (*P* > 0.050). Severe recent stress increased the vulnerability to IDs among CA-HIV (*P* = 0.001). We did not observe any gene–environment effect on vulnerability to IDs in this population. These findings support the currently held opinion that polymorphisms at single genetic loci only contribute a very small effect to the genetic vulnerability to IDs.

## Introduction

Poor mental health has been recognized as a neglected global health burden, particularly for children and adolescents (1). Globally, 10–20% of children suffer from life-related disabilities arising from mental health disorders, including psychiatric disorders, general psychological distress, and emotional and behavioral problems (2). However, studies on mental health among children and adolescents have lagged considerably behind that of adults, especially in the African continent (3). Children and adolescents with human immunodeficiency virus (CA-HIV) represent one of the groups with a significant burden of mental health disorders (4, 5). A study conducted in the United States reported a higher prevalence of psychiatric cases in children with HIV compared to the general pediatric population (6.17 vs. 1.7 cases per 1,000 person-years) (6). Evidence for the adverse effects of HIV infection on mental health has also been found in sub-Saharan African populations (7), with a systematic review of 14 studies indicating a 25% prevalence for any psychiatric disorder and a 30–50% prevalence of psychological distress or emotional or behavioral difficulties in adolescents with HIV (8). One of the groups of mental illness that CA-HIV suffer from is internalizing disorders (IDs; depressive and anxiety disorders) (5, 9, 10).

Internalizing disorders are most often characterized by quiet, internal distress, which may be described as “intropunitive,” rather than overt, socially negative, or disruptive behavior that characterizes externalizing disorders (11). The most prevalent and commonly diagnosed IDs include depression and anxiety, with studies from both Africa and the United States reporting ranges between 4–72 and 5–82%, respectively (5, 12). In Uganda, an epidemiological study, on which this analysis is based, reported the prevalence of IDs as 11.5% and of major depressive disorder as 3.9% and any anxiety disorder as 9.0% (5).

The risk factors for IDs in CA-HIV are multifactorial and include the direct and indirect effects of HIV neurotoxicity (13). Similarly, environmental risk factors such as bereavement, extreme poverty, food insecurity, and deprivation of basic needs have also been implicated in IDs (14, 15). These environmental factors predispose to acute/recent and/or chronically stressful life conditions, which shape the onset, temporal course, and consequences of depressive and anxiety disorders (16, 17). IDs have been associated with poor school performance, impaired social relationships and reduced drug adherence, risky sexual behavior as well as impaired quality of life (14, 15).

A familial history of psychiatric illness among children and adolescents has been demonstrated to be associated with IDs, lending credence to the role of genetic factors in determining internalizing disorder risk (18). Genetic polymorphisms within the neurotransmitter and neuropeptide signaling systems—for instance, brain-derived neurotropic factor (rs6265, rs10835210, and rs11030107) (19), dopamine transporter gene (20), monoamine oxidase A (21), and serotonin transporter (22) genes, have been associated with IDs in healthy children and adolescents in the western world. Albeit that the psychiatric–genomic data in African populations is limited, a study undertaken in Uganda (23) reported a significant association between polymorphisms of the serotonin transporter gene and suicidality among adults living with HIV/AIDS. Notably, the only study to date that has investigated genetic factors associated with psychiatric disorders among CA-HIV has reported a significant interaction between recent stress and the *S*_*A*_ haplotype of the serotonin-transporter-gene-linked polymorphic region, which was associated with higher odds of being an ID case (24).

On the other hand, genetic studies conducted in non-HIV-infected populations have reported associations between genes within the stress response-hypothalamic-pituitary-adrenal axis (HPA) and vulnerability to depression and anxiety. In particular, vulnerability to IDs has been associated with single-nucleotide polymorphisms (SNPs) within both the FK506 binding protein 51 (*FKBP5*) gene, which encodes a glucocorticoid receptor (GR) co-chaperone that is involved in the HPA axis negative feedback (25, 26), and the nuclear receptor subfamily-3 group-C member-1 (*NR3C1*) gene, which encodes the GR protein (27, 28). SNPs such as rs3800373, rs9296158, rs1360780, rs9470080, rs4713916, rs3000377, and rs47139611 in *FKBP5* (25, 26) and rs414232, rs6195, rs6189, rs10482605, and rs6190 in *NR3C1* (27, 28) are postulated to interact with early trauma or childhood adversity to predict negative outcomes later in life, such as vulnerability to IDs (29, 30). The minor *C* allele of the rs10482605 polymorphism, located within the promoter region of *NR3C1*, is associated with a reduced expression of the GR protein and has been inversely associated with the liability to develop IDs in non-HIV-infected subjects (27, 28). Similarly, rs1360780, a SNP located in an enhancer region 488 bp away from a glucocorticoid response element in intron 2 of *FKBP5*, moderates the GR-mediated expression of FKBP5 (25, 26). Several groups reported an association between *FKBP5* variants and IDs using American, German, and Polish non-HIV cohorts (31–33). However, other studies involving Spanish, Swedish, and Italian populations failed to replicate these findings (34–36). Whether common variants in *FKBP5* are associated with vulnerability to IDs thus remains inconclusive.

Additionally, these studies have been conducted among non-HIV-infected adults, primarily in populations of European ancestry. Therefore, the prevalence of these SNPs and their association with vulnerability to IDs (depression and anxiety) in an African population of CA-HIV is unclear. We investigated GR and FK506 binding protein genotypes and stress as predictors of IDs among CA-HIV from Kampala and Masaka districts. Finally, the role of gene-environment interactions in predisposing to IDs was also explored.

## Materials and Methods

### Study Design

This was a nested case-control study utilizing archived whole blood samples collected during a Medical Research Council/Department for International Development (MRC/DFID)-funded project entitled “Mental health among children and adolescents living with HIV/AIDS in Kampala and Masaka districts of Uganda” (CHAKA study). The aim of the CHAKA study was to investigate the epidemiology of psychiatric disorders among CA-HIV in Uganda and the implications of these for service provision (5). The present study investigated the association between stress on the one hand and selected stress hormone pathway gene polymorphisms on the other with IDs among CA-HIV.

### Study Participants and Site

The CHAKA parent study enrolled a total of 1,339 children and adolescents [855 children (5–11 years) and 484 adolescents (12–17 years)] from two HIV clinics in Kampala (Joint Clinic Research Centre and Nsambya Home Care) and three HIV clinics in Masaka (The Aids Support Organization-Masaka, Kitovu Mobile Clinic, and Uganda Cares). The participants were recruited and assessed for psychiatric disorders (including IDs) and socio-demographic factors at three time points (baseline and at months 6 and 12 months) by trained psychiatric nurses and psychiatric clinical officers between December 2014 and August 2016 (5). All study participants were on antiretroviral treatment (ART). The current study is comprised of a total of 736 cases and matched control participants [389 children (5–11 years) and 307 adolescents (12–17 years)] who were recruited into the CHAKA study.

### Inclusion and Exclusion Criteria

The following were the inclusion and exclusion criteria for the CHAKA study:

**Inclusion criteria**: (i) CA-HIV (with children defined as aged between 5 and 11 years and adolescents defined as aged between 12 and 17 years), and (ii) able to speak English or Luganda (the local language spoken in the study areas) and expected to remain in the study area for the subsequent 12 months.

**Exclusion criteria**: (i) concurrent enrollment in another study, (ii) sick and in need of immediate medical attention, and (iii) those unable to understand the study instruments for whatever reason.

The current study included all children (aged 5–11 years) and adolescents (aged 12–17 years) with IDs and their case-matched controls (children and adolescents without any psychiatric disorder). Details about the prevalence of psychiatric disorders diagnosed in the CHAKA study, including internalizing disorders considered in the current study, have been included in Supplementary Table 1.

### Selection of Cases and Controls

The selection of cases and controls was as described elsewhere (37). Briefly, the cases were children/adolescents who had any IDs. All cases at baseline were ascertained, and the cases were then stratified by site (one of two sites), sex, age category (one of three categories), and socio-economic status (SES; one of three SES categories). This resulted in a total of 36 strata (2 × 2 × 3 × 3). In each stratum, the number of cases was ascertained (e.g., for males in site 1 in the youngest age category and the lowest SES group, there were nine cases). An equal number of controls (children/adolescents without any psychiatric disorder) were then randomly sampled from the stratum concerned (so for males in site 1 in the youngest age category and the lowest SES group, nine controls were sampled). Thus, the controls were frequency matched to the cases on site, sex, age, and SES.

### Power Calculation

We conducted a *post-hoc* power analysis for this study. The sample size calculation was done using an online calculator (http://osse.bii.a-star.edu.sg/calculation1.php). The default setting for a case–control study was used, with significance level of 5%, minor allele frequency in cases of 15%, minor allele frequency in controls of 7%, and ratio of cases to controls at 1:1. Given 309 cases and an equal number of controls with complete genotypic data, our study achieved a power of 89%.

### Assessment of Psychiatric Disorders and Socio-Demographic Factors

The details of the procedures has been published elsewhere (37). Psychiatric disorders, including IDs, were assessed using the Children and Adolescent Symptoms Invetory-5 (CASI-5) (Gadow and Sprafkin, 2013). The CASI-5 has been locally adapted for use in Uganda (38). The CASI-5 was administered by trained psychiatric nurses and psychiatric clinical officers at two time points (baseline and 12 months). Individual CASI-5 items are rated on a four-point frequency of occurrence scale ranging from never (0) to very often (3). The socio-demographic characteristics for both the participants and their caregivers were collected using a socio-demographic proforma developed by the Mental Health Section of the MRC/Uganda Virus Research Institute and LSHTM Uganda Research Unit. The socio-economic status was generated from a scale of nine household items (car, motorcycle, refrigerator, electricity, bicycle, radio, telephone, cupboard, and flask). Each item was weighted in the respective order, with a car carrying a maximum weight of 9 and a flask a minimum weight of 1. A total score of the items was generated, whose median cutoff of 13 was used to classify low and high SES. A score <13 was classified as low SES, while that >13 was classified as high SES.

### Assessment of Chronic and Recent Stress

The chronic and recent stress classes used in this study were generated by Kalungi et al. (37). Briefly, social disadvantage was used as a proxy of chronic stress, with a composite index constructed based on the following variables: orphanhood, food availability, study site, and level of education of the caregiver. Hierarchical cluster analysis (HCA) was used to generate the different cutoff points for each of the chronic stress classes: mild with chronic stress scores of 0–1.375, moderate with scores ranging from 1.375 to 2.375, and severe with scores >2.375. A composite index for recent stress was constructed from the following variables: caregiver mental state, child–caregiver relationship, and HIV symptoms. Three recent stress classes were generated using HCA, i.e., mild with scores <0.362, moderate with scores between 0.362 and 0.622, and severe with scores >0.622.

### DNA Extraction

Genomic DNA was extracted from a whole-blood specimen for each participant using the Qiamp mini DNA extraction kit (Qiagen GmbH, Germany), following the instructions of the manufacturer. Extracted DNA was quantified by ultraviolet spectrophotometry on NanoDrop 1000 spectrophotometer V3.7 (Thermo Fisher Scientific, Wilmington, MA). The DNA was subsequently diluted to 5 ng/μl, and 5 μl of each of the DNA samples was run on 1% agarose gel to assess the quality. The extracted DNA was then stored for ~3 months at −20°C while awaiting further molecular analysis.

### Kompetitive Allele-Specific PCR Genotyping

The Kompetitive Allele-Specific PCR (KASP) genotyping of rs10482605 (within *NR3C1*) and rs1360780 (within *FKBP5*) polymorphisms was conducted by LGC Genomics Ltd., United Kingdom. Briefly, the PCR reaction was carried out in 10 μl of reaction mixture containing 5 μl of 2X KASP master mix, KASP assay mix (primers) at 0.14 and 5 μl (5 ng/μl) of genomic DNA. The DNA sample was diluted to a final concentration of 5–10 ng per reaction. All reagents were vortex-mixed prior to use. The cycling conditions were as follows: one cycle of hot start activation at 94°C for 15 min, followed by 10 cycles of denaturation, annealing, and extension at 94°C for 20 s and 61–55°C for 60 s (dropping 0.6°C per cycle), respectively. A further 26 cycles were performed, consisting of denaturation at 94°C for 20 s and annealing and extension at 55°C for 60 s (39). Distilled water and PCR mixture without the DNA template were included on each genotyping plate as negative controls. A difference in fluorescent signal intensity between the presence and absence of template DNA allowed improved confidence in the validity of the genotyping results. Individual samples that failed to genotype for a particular SNP were removed from the analysis.

### Statistical Analysis

Statistical analyses were conducted using Stata 15 (StataCorp, TX, USA). The socio-demographic characteristics (including SES) were dichotomized and described between cases and controls. The socio-economic status and chronic and recent stress were measured as described by Kalungi et al. (37). Hierarchical cluster analysis was used to generate the different cutoff points for each of the chronic and recent stress classes (mild, moderate, and severe). Chi-square tests were used to assess the differences between the socio-demographic characteristics according to ID status at baseline (cases vs. controls). The allele frequencies of the studied genes were tested for Hardy–Weinberg equilibrium (HWE), and the distribution of genotype in cases and controls was compared using chi-square tests. Any possible relationship between polymorphisms in each gene and ID was investigated using chi-square tests. Logistic regression models were used to investigate the association between recent stress, chronic stress, genotypes, and IDs and to calculate the odds ratio and 95% confidence intervals. In these models, recent and chronic stress were included as predictor variables, SNPs were entered as covariates, and ID case-control status was the binary outcome variable. The interaction effect of chronic stress/recent stress on the association between the polymorphisms (rs10482605 and rs1360780**)** and IDs was tested using a likelihood ratio test.

### Ethical Considerations

The present study was nested in a parent study (37), which obtained ethical and scientific clearance from the Uganda Virus Research Institute Science and Ethical Committee (#GC/127/15/06/459) and the Uganda National Council of Science and Technology (#HS 1601). Written informed consent was obtained from all the caregivers for their children/adolescents to participate in the study and for a blood specimen to be drawn from their children/adolescents for genetic analyses. The adolescents provided further written informed assent to participate in the study. The study participants diagnosed with significant psychiatric problems were referred to the mental health units at Entebbe and Masaka government hospitals. Personal identifiers and data were kept under lock and key and were only available to the study team. Administrative clearance was obtained from the Uganda Virus Research Institute—MOH for the use of the archived samples.

## Results

### Socio-Demographic Characteristics of the Study Participants

A total of 736 participants (389 children and 307 adolescents) were enrolled into this study from the Kampala and Masaka districts. Out of the 736, about 44% were from Kampala, and 56% were recruited from Masaka. The subjects were purposively selected and age- and sex-matched based on the availability of 368 presenting with at least one ID, hence giving both controls and cases equal representation of 50% (368/736 each). The rs10482605 polymorphism was assessed in 624 (309 cases and 315 controls) participants with complete data, while 685 participants (335 controls and 350 cases) had complete data for inclusion in the rs1360780 polymorphic analysis. The socio-demographic characteristics did not differ by disease status (Table 1). The socio-demographic characteristics were analyzed as described in the “Statistical Analysis” section.

**Table 1:** Distribution of socio-demographic characteristics between cases and controls.

**Characteristics**	**Controls, ***n*** (%)**	**Cases, ***n*** (%)**	**Total, ***n*** (%)**	***P***
**Gender**				
Female	207 (56.40)	186 (50.54)	393 (53.47)	0.111
Male	160 (43.60)	182 (49.46)	342 (46.53)	
**Age category**				
Adolescents	149 (42.45)	158 (45.80)	307 (44.11)	0.374
Children	202 (57.55)	187 (54.20)	389 (55.89)	
**Educational level**				
No formal education	9 (2.45)	4 (1.09)	13 (1.77)	0.371
Pre-primary	323 (88.01)	325 (88.80)	648 (88.40)	
Secondary	35 (9.54)	37 (10.11)	72 (9.82)	
**Socio-economic status**				
High	197 (53.53)	207 (56.25)	404 (54.89)	0.459
Low	171 (46.47)	161 (43.75)	332 (45.11)	
**Study site**				
Kampala	165 (44.84)	156 (42.39)	321 (43.61)	0.504
Masaka	203 (55.16)	212 (57.61)	415 (56.39)	

*n, number of participants; %, percentage; children, 5–11 years; adolescents, 12–17 years*.

### Distribution of the rs10482605 and rs1360780 Minor Allele Frequencies and Genotypes Between Cases and Controls

The comparison between minor allele frequencies of both rs10482605 (within *NR3C1*) and rs1360780 (within *FKBP5*) SNPs in the studied population and other ethnicities is shown in Table 2. The minor allele frequencies of rs10482605 and rs1360780 polymorphisms were 0.121 and 0.396, respectively, in this study population.

**Table 2:** Genome location and minor allele frequency of the rs10482605 and rs1360780 single nucleotide polymorphisms among different populations.

**Genome location**				**Minor allele frequency**					
**Gene region**	**Xsome**	**Nucleotide change**	**DBSNP Ref**	**1000G-Global**	**1000G-African**	**Est**	**Swe**	**Sib**	**Ug**
NR3C1-promoter	Chr5	A>G	rs10482605	0.10	0.08	0.12	0.13	0.50	0.12
FKBP5-intron 2	Chr6	T>C	rs1360780	0.33	0.42	0.26	0.28	0.24	0.39

*Xsome, Chromosome; Est, Estonian; Swe, Sweden; Sib, Siberia; Ug, Uganda; DBSNP Ref, database single nucleotide polymorphism reference (allele frequency adopted from 1,000 genomes; https://www.ncbi.nlm.nih.gov/snp/)*.

#### Frequencies and Genotype Distribution of NR3C1 rs10482605

The rs10482605 polymorphism was assessed in 624 (309 cases and 315 controls) participants with complete data. In particular, out of 315 participants without IDs, five carried the homozygous minor *GG* genotype for the rs10482605 polymorphism, while 60 and 250 participants carried the heterozygous *GA* and wild-type *AA* forms, respectively. Similarly, out of 309 subjects with IDs, four possessed the homozygous minor *GG* genotype of the rs10482605 polymorphism; 73 and 232 were heterozygous and homozygous wild type, respectively. In comparison, 44.44 and 55.56, 54.89 and 45.11, and 48.13 and 51.87% of cases and controls possessed the mutant, heterozygous, and wild type of the *NR3C1* genotypes, respectively (Table 3). According to the results of a chi-square test, there was no significant difference in the frequencies and genotype distribution of the rs10482605 polymorphism between cases and control populations (*P* > 0.369). The frequency of the minor *G* allele in the rs10482605 polymorphic site was 0.121 among the population studied (Table 2). The genotype distribution for the subjects did not significantly deviate from HWE (*P* = 0.9595).

**Table 3:** Distribution of rs10482605 and rs1360780 genotypes between cases and controls.

**Genotypes**	**Cases**,	**Controls**	***P***
	***n*** **(%)**	***n*** **(%)**	
rs10482605	*N* = 309	*N* = 315	
Wild type (*AA*)	232 (48.13)	250 (51.87)	0.369
Heterozygous (*GA*)	73 (54.89)	60 (45.11)	
Mutant (*GG*)	4 (44.44)	5 (55.56)	
rs1360780	*n* = 350	*n* = 335	
Wild type (*CC*)	133 (52.36)	121 (47.64)	0.492
Heterozygous (*CT*)	166 (51.88)	154 (48.13)	
Mutant (*TT*)	51 (45.95)	60 (54.05)	

*n, number of participants; %, percentage*.

#### Frequencies and Genotype Distribution of FKBP5 rs1360780

A total of 685 participants (335 controls and 350 cases) had complete data for inclusion in the rs1360780 polymorphic analysis, as shown in Table 2. The number of individuals carrying the homozygous minor *TT*, heterozygous *CT*, and wild-type *CC* genotypes were 60, 154, and 121, respectively, among the controls and 51, 166, and 133, respectively, among the cases (Table 3). The genotype frequencies were 54.05 and 45.95%, 48.13 and 51.88%, and 47.64 and 52.36% for homozygous minor *TT*, heterozygous *CT*, and wild-type *CC* among controls and cases, respectively (Table 3). There was no significant difference observed in the frequency and distribution of the different genotypes between the cases and controls for the rs1360780 polymorphism using the chi-square test (*P* = 0.492). In addition, the genotype distribution for subjects did not significantly deviate from the HWE as evidenced by the non-significant difference between the observed and expected alleles and genotype values of the rs1360780 polymorphisms (*P* = 0.545). The minor allele *T* frequency in this population was 0.396 (Table 2).

### Genotypic and Environmental Predictors of IDs in CA-HIV From Kampala and Masaka Districts—Uganda

A total of 309 cases had complete data for inclusion in the analyses of the association between IDs and the potential risk factors of the investigated SNPs and environmental factors (acute and chronic stress). None of the allelic forms of the SNPs studied had any significant influence on the increased likelihood of developing IDs (Table 4). Using logistic regression analysis, the decreased risk of developing IDs in individuals with the *G* minor allele of the rs10142605 was not significant (*P* = 0.143) compared to individuals possessing the wild-type form. In individuals carrying the homozygous minor *T* allele of the rs1360780 variant, the odds of developing IDs was likewise non-significantly reduced (*P* = 0.329). Recent stress (specifically severe recent stress), as opposed to chronic stress, significantly increased the likelihood of developing IDs in CA-HIV as shown in Table 4 (*P* = 0.001). Moderate chronic stress appears to protect against IDs (*P* = 0.026).

**Table 4:** Association between chronic and recent stress and internalizing disorders (IDs) in children and adolescents living with human immunodeficiency virus from Kampala and Masaka Districts—Uganda.

**Predictors of IDs**	***n*** **= 309**	
	**OR (95% CI)**	***P***
**Environmental factors**		
Chronic stress		
Mild	1.0	Reference
Moderate	0.69 (0.49–0.95)	0.025^a^
Severe	0.92 (0.61–1.37)	0.673
**Recent stress**		
Mild	1.0	Reference
Moderate	1.07 (0.76–1.52)	0.687
Severe	1.85 (1.29–2.65)	0.001^a^
**Genotype**		
*NR3C1* rs10482605		
Wild type (*AA*)	1.0	Reference
Heterozygous (*GA*)	1.34 (0.91–1.99)	0.140
Mutant (*GG*)	0.73 (0.19–2.81)	0.649
***FKBP5*** **rs1360780**		
Wild type (*CC*)	1.0	Reference
Heterozygous (*CT*)	1.08 (0.76–1.55)	0.636
Mutant (*TT*)	0.78 (0.48–1.27)	0.329

*CI, confidence interval; OR, odds ratio*.

a *Statistically significant*.

We interrogated the data to further assess whether individuals carrying the polymorphic risk variants (we predicted that these participants would be more vulnerable to the effect of environmental adversity) are more likely to develop psychopathology. All the environmental adversities assessed were summed up and dichotomized as chronic or recent stress as described. Using likelihood ratio analysis, there was no significant association between gene–environment interactions and susceptibility to IDs (*P* > 0.050), as shown in Table 5.

**Table 5:** Gene-environment interaction.

**Gene-environment interaction**	**OR (95% CI)**	***P***	**Overall** ***P***
**rs10482605 × chronic stress (*****N*** **= 624)**			
*AA*—mild chronic stress	1.0	Reference	0.340
*GA*—moderate chronic stress	2.56 (105–6.24)	0.039	
*GA*—severe chronic stress	1.36 (0.49–3.76)	0.549	
*GG*—moderate chronic stress	0.765 (0.19–30.6)	0.887	
*GG*—severe chronic stress	1.09 (0.35–33.95)	0.959	
**rs10482605 × recent stress (*****N*** **= 624)**			
*AA*—mild recent stress	1.0	Reference	0.553
*GA*—moderate recent stress	0.828 (0.33–2.07)	0.687	
*GA*—severe recent stress	1.75 (0.64–4.75)	0.270	
*GG*—moderate recent stress	0.939 (0.06–13.9)	0.964	
*GG*—severe recent stress	1.00	omitted	
**rs1360780 × chronic stress (*****N*** **= 685)**			
*CC*—mild chronic stress	1.0	Reference	0.743
*CT*—moderate chronic stress	1.41 (0.673–2.962)	0.361	
*CT*—severe chronic stress	1.59 (0.629–4.039)	0.325	
*TT*—moderate chronic stress	0.83 (0.297–2.336)	0.728	
*TT*—severe chronic stress	1.2 (0.359–4.01)	0.767	
**rs1360780 × recent stress (*****N*** **= 685)**			
*CC*—mild recent stress	1.0	Reference	0.2628
*CT*—moderate recent stress	2.44 (1.086–5.50)	0.031	
*CT*—severe recent stress	1.17 (0.52–2.63)	0.701	
*TT*—moderate recent stress	1.41 (0.487–4.101)	0.523	
*TT*—severe recent stress	0.894 (0.286–2.795)	0.848	

*CI, confidence interval; OR, odds ratio*.

## Discussion

This study determined the allele and genotype frequencies of SNPs in *NR3C1* (rs10482605) and *FKBP5* (rs1360780) and examined the relationship between these SNPs, environmental stress, and vulnerability to IDs in CA-HIV in Kampala and Masaka districts in Uganda. The minor allele frequencies of rs10482605 and rs1360780 polymorphisms were 0.121 and 0.396, respectively, among this study population. The rs1360780 minor allele frequency of 0.396 is similar to the 0.39–0.45 documented in African Americans (40) but somewhat higher than those in Estonian, Swedish, and Siberian populations. Similarly, the rs10482605 minor allele frequency of 0.121 is higher than the global and African minor allele frequencies reported in the 1,000 Genomes, equal to those in the Estonians (0.12) and Swedish (0.13) but lower than those in the Siberian (0.50) populations. The inconsistencies in polymorphism frequencies might be due to genetic ancestry-related differences and reinforces the importance of studying genetically driven susceptibility to disorders in diverse populations. Our result show that *AA* was the most prevalent genotype of the rs10482605 SNP with a frequency of 77.2%, whereas the frequency of the minor *GG* genotype was 1.44%. This finding mirrors the frequencies of 93.1, 1.6, and 5.3% for *AA, AG*, and *GG*, respectively, reported among the Chinese population (41). However, this result conflicts with a previous study conducted in a German population where genotype frequencies of 67, 27.6, and 4.4% for *GG, AG*, and *AA*, respectively, were reported (42). These similarities and differences are probably due to differences in ancestry. With regards to the rs1360780 SNP in our sample, the following genotype frequencies of 37.0, 46.7, and 16.3% for *CC, CT*, and *TT*, respectively, were reported. Schneider et al. (42) also reported similar findings among the German population.

The distribution as well as the role of the genotypes discussed above in vulnerability to IDs was investigated using a case–control study design. Overall, there was no significant difference in genotype distribution/frequencies between case and control populations for both rs10482605 and rs1360780. Remarkably, there was no association between the carriage of the rs10482605 polymorphic *G* risk allele in this population and vulnerability to IDs, consistent with previous studies among child psychiatric population (43) and the Chinese Han population (41). However, the rs10482605 polymorphism, which is in linkage disequilibrium with rs6198, is associated with increased GR beta mRNA stability, and previous authors have reported the indirect effects of this gene with the IDs of depression and anxiety (26, 42). Possession of the rs10482605 risk allele was associated with childhood mood disorders in a mixed Canadian population (44) and recurrent major depression in Belgian and Swedish populations (43). Recently, Womersley et al. in South Africa, reported that the rs10482605 risk allele was associated with increased sensitivity to anxiety among adolescents (45). In this study, the proportion of participants carrying the *TT* polymorphic risk allele of rs1360780, which is located in intron 2 of *FKBP5*, did not differ significantly between cases and controls. Schneider et al. in a study among pregnant women in Germany, observed no association between polymorphisms in the *FKBP5* gene and depressive scores (42). Other researchers, however, have reported conflicting results. Menke et al. reported a significant association between the *TT FKBP5* alleles and depression among an adult German population (46). Binder et al. in another German study, reported a significant association between polymorphisms in the *FKBP5* gene and the recurrence of depressive episodes and response to antidepressants (47). In a European study conducted among depression treatment-resistant adolescents, *FKBP5* rs1360780 was associated with suicide, a distal correlate of depression (48). It is plausible that the rs1360780 and rs10482605 polymorphisms may not be involved in the pathophysiology of IDs in CA-HIV in the Ugandan population.

There are a number of possible reasons for these discrepant results. Firstly, on a more general conceptual level, the IDs of depression and anxiety have a multi-factorial etiology that includes biological (including genetic) and environmental risk factors (5, 9, 49, 50). It is now believed that the predisposition to IDs, such as depression, is determined by the coordinated action of many genes and their interaction with each other and with diverse environmental factors (51). We did not observe any gene–environment effect on vulnerability to IDs in this population. It is also likely that each gene, by itself, makes a relatively small contribution to the pathogenesis of the disease (52). As a result of this and as shown in this study, it has been difficult to replicate genetic associations. Shadrina et al. (51), in a recent review article, highlighted the challenges of replicating genetic association studies despite the fact that a large number of the associations between genes and different clinical depression variants and depressive sub-phenotypes have been published.

Secondly, another reason for the discrepant results could be methodological; the sample size for this study may have been inadequate to identify the small contribution that each of these SNPs is hypothesized to contribute to the risk of the IDs of depression and anxiety. Hence, this study was probably not adequately powered to test the association between these SNPs and IDs. This observation is supported by recent efforts to identify common genetic variants associated with depression in genome-wide association studies (GWAS), which have previously been unsuccessful largely because of inadequate samples. In a recent successful GWAS, in order to maximize the sample size, Howard et al. (49), in a meta-analysis, used data on 807,553 individuals (246,363 cases and 561,190 controls) and were able to identify 102 independent variants associated with depression. Lastly, another factor that may have contributed to the absence of an association between the investigated SNPs and IDs is the differences in participant ancestry between this and previous studies. While this study was undertaken among respondents of African ancestry, all the previous studies that reported positive results were of Caucasian ancestry (46–48). It is possible that it is not the rs1360780 and rs10482605 SNPs themselves that increase the risk for IDs but rather another variant or variants that are in close linkage disequilibrium (53). Ancestry-based differences in haplotype structure may thus account for discrepant findings across different study groups. Furthermore, ancestry may also affect the degree to which genetic variants play a role in determining local and more distal gene expression (54).

In this study, we found an association between IDs and both recent and chronic stress. The odds of IDs increased with increasing severe recent stress and decreased with moderate chronic stress. This finding is consistent with previous studies where recent stress has been associated with the IDs of depression (9, 55) and anxiety (56). Interestingly, the odds of developing IDs decreased with moderate chronic stress in CA-HIV. The seemingly protective effect of moderate chronic stress in this study could not be easily explained but could be attributed to physiological and biochemical cross-adaptations. Moderate chronic stress has also been shown to be associated with alleviation of depressive symptoms in open water swimmers (57). However, a recent study in animal models showed that, in young adult female mice, chronic stress resulted in decreased anxiety-like behavior and enhanced cognitive performance, whereas in old female mice it led to weight loss, dysregulated locomotion, and memory impairment, suggesting that the effect of chronic stress on behavior is age dependent (58). These results may have underlined the findings in our study as the study respondents were relatively very young (aged between 5 and 17 years). Further empirical evidence is warranted to understand the mechanisms underpinning the observed positive clinical effect. This could revolutionize the extant therapies and lead to potential non-drug pathways to recovery from IDs.

In conclusion, the results from this study seem to give credence to the stress-vulnerability diathesis of depression as postulated by Ising and Holsboer (59), where acute and chronic stress were both associated with IDs. However, in departure from the stress-vulnerability diathesis of Ising and Holsboer (59), moderate chronic stress among young respondents in this study seemed to be protective against IDs. These results, however, need to be replicated by further studies. Additionally, while the results from this study showed the contribution of psychosocial stressors on ID vulnerability, they did not show a contribution of genetic factors to vulnerability as originally hypothesized. We interrogated the data to further assess whether individuals carrying the polymorphic risk variants (we predicted that these participants would be more vulnerable to the effect of environmental adversity) were more prone to developing a psychopathology. Using logistic regression analysis, there was no significant association between gene–environment interaction and susceptibility to IDs.

The study has some limitations: (1) recall bias: the number and nature of adverse environmental episodes in the past month were obtained retrospectively from caregivers and participants (in the parent CHAKA study) by a psychiatric clinician during history taking. Studies have shown that the retrospective reporting of childhood trauma is valid (60) and is not influenced by psychiatric state at the time of the report (61). In this regard, it is noteworthy that all the participants were interviewed using standardized tools and by trained psychiatric clinicians; (2) study design: the study sample was small, given the relatively small contribution that each SNP makes to the risk of depression. Furthermore, our study was limited to two SNPs. Though these were selected based on previous publications, the more comprehensive, and expensive, genome- or transcriptome-wide approaches are better suited to investigating the role of multiple genetic variants in complex disorders such as IDs; and (3) control group composition: we acknowledge that factors such as ART could associate with ID status and influence the study findings. We also acknowledge that HIV positivity status constitutes a chronic stressor through the persistent disturbance of the immune, cardiovascular, metabolic, and behavioral homeostasis (62). However, any potential confounding effect of HIV diagnosis on the qualification of chronic stress in this study is buffered out by the fact that both cases and controls were HIV-positive. Nonetheless, this study would be preferred among a general population; but, since it was nested in a parent study which investigated psychiatric disorders among Ugandan HIV+ children and adolescents, the clinical data and sample available to us was in this cohort.

## Conclusion

Neither of the SNPs investigated in this study was significantly associated with vulnerability to IDs. However, as previously reported by others, severe recent stress was a significant risk factor of IDs. Additionally, in this study, moderate chronic stress seemed to be a protective factor against IDs, an observation that has only been previously reported in animal models. We did not observe any gene–environment effect on vulnerability to IDs in this population.

## Recommendation

There is a need to undertake a GWAS to illuminate the role of genetic factors in the risk of IDs among respondents of African ancestry. The observation of the possible protective role against IDs of moderate chronic stress among relatively young respondents observed in this study warrants further inquiry.

## Data Availability Statement

All information pertaining to participants and samples remain confidential with access limited to research team. However, upon request data from the MRC/UVRI and LSHTM Uganda research unit is currently accessed under a data sharing policy via http://www.mrcuganda.org/sites/default/files/publications/MRC_UVRI_Data_Sharing_Policy_December2015.pdf. Requests to access these datasets should be directed to http://www.mrcuganda.org/sites/default/files/publications/MRC_UVRI_Data_Sharing_Policy_December2015.pdf.

## Ethics Statement

The studies involving human participants were reviewed and approved by Uganda Virus Research Institute (UVRI) Science and Ethical Committee (# GC/127/15/06/459), the Uganda National Council of Science and Technology (# HS 1601). Written informed consent was obtained from all the caregivers for their children/adolescents to participate in the study and for a blood specimen to be drawn from their children/adolescents for genetic analyses. Adolescents provided further written informed assent to participate in the study. Personal identifiers and data were kept under lock and key and were only available to the study team. Administrative clearance was obtained from the UVRI-MOH for the use of the archived samples. Written informed consent to participate in this study was provided by the participants' legal guardian/next of kin.

## Author Contributions

TJO and AK conceived and conducted the study and drafted the first manuscript. WJS carried out the statistical analysis. DM, JSW, and EK supervised and edited the manuscript. All authors reviewed and approved the manuscript for publication.

## Funding

This study was funded by a grant from the Alliance for Global Health Sciences, which was awarded to AK (Grant No. 50288/N7145). The parent study investigating mental health among children and adolescents living with HIV/AIDS in Kampala and Masaka districts of Uganda (CHAKA study) was funded by a Medical Research Council/Department for International Development African Research Leader award to EK (Grant No. MR/L004623/1).

## Conflict of Interest

The authors declare that the research was conducted in the absence of any commercial or financial relationships that could be construed as a potential conflict of interest.

## Publisher's Note

All claims expressed in this article are solely those of the authors and do not necessarily represent those of their affiliated organizations, or those of the publisher, the editors and the reviewers. Any product that may be evaluated in this article, or claim that may be made by its manufacturer, is not guaranteed or endorsed by the publisher.
