# Supplementary material for: Association of Stress, Glucocorticoid Receptor, and FK506 Binding Protein Gene Polymorphisms With Internalizing Disorders Among HIV-Infected Children and Adolescents From Kampala and Masaka Districts—Uganda
Source: Front Pediatr. 2021 Oct 26;9:666426. doi: 10.3389/fped.2021.666426 (PMC8576357; doi:10.3389/fped.2021.666426)
Supplement: Supplementary file 1 [file Table_1.pdf]

Supplementary Table 1: Prevalence of psychiatric disorders among children and adolescents attending the CHAKA Study (adopted from Kinyanda *et al.*, 2019 [5])

Psychiatric disorder	Caregiver report CASI-5			Youth self-report YI-R	Either caregiver or youth self-report Either by CASI-5 or YI-R	
	Children (A)	Adolescents (B)	Total (C)	Adolescents (D)	Adolescents (Either B or D)	Total (Either C or D)
Any psychiatric disorder	117 (13.7%) (11.5–16.2)	70 (14.5%) (11.6–17.9)	187 (13.9%) (12.2–15.9)	52 (10.7) (8.2–13.8)	115 (23.8%) (20.2–27.8)	233 (17.4%) (15.4–19.5)
Any behavioral disorder[a]	66 (7.7%) (6.1–9.7)	31 (6.4%) (4.5–9.0)	97 (7.2%) (6.0–8.8)	33 (6.8%) (4.8–9.4)	60 (12.4%) (9.7–15.6)	128 (9.6%) (8.1–11.2)
Oppositional defiant disorder	31 (3.6%) (2.6–5.1)	14 (2.9%) (1.7–4.8)	45 (3.4%) (2.5–4.5)	14 (2.9%) (1.7–4.8)	27 (5.6%) (3.8–8.0)	58 (4.3%) (3.4–5.5)
Conduct disorder	13 (1.5%) (0.8–2.6)	9 (1.7%) (1.0–3.5)	22 (1.6%) (1.1–2.5)	14 (2.9%) (1.7–4.8)	23 (4.7) (3.2–7.1)	39 (2.9%) (2.1–4.0)
Attention deficit hyperactivity disorder	40 (4.7%) (3.4–6.3)	18 (3.7%) (2.3–5.8)	58 (4.3%) (3.4–5.6)	15 (3.1%) (1.9–5.1)	31 (6.4%) (4.5–9.0)	71 (5.3%) (4.2–6.6)
Any emotional disorder[b]	66 (7.7%) (6.1%)	47 (9.7%) (7.4–12.7)	113 (8.4%) (7.1–10.0)	47 (9.7%) (7.3–12.7)	88 (18.2%) (15.0–21.9)	**154 (11.5%) (9.9–13. 3)**
Any anxiety disorder	49 (5.7%) (4.3–7.5)	29 (6.0%) (4.2–8.5)	78 (5.8%) (4.6–7.2)	49 (10.1%) (7.7–13.1)	71 (14.7%) (11.8–18.1)	**120 (9.0%) (7.5–10.6)**
Generalized anxiety disorder	17 (1.9%) (1.2–3.2)	10 (2.1%) (1.1–3.8)	27 (2.0%) (1.3–2.9)	26 (5.4%) (3.7–7.8)	35 (7.2%) (5. 2–9.9)	52 (3.9%) (3.0–5.1)
Social anxiety disorder	10 (1.2%) (0.6–2.1)	8 (1.6%) (0.8–3.2)	18 (1.34%) (0.8–2.1)	*	34 (7.0%) (5.1–9.7)	44 (3.3%) (2.4–4.4)
Separation anxiety disorder	28 (3.3%) (2.3–4.7)	18 (3.7%) (2.3–5.8)	46 (3.4%) (2.6–4.5)	10 (2.1%) (1.1–3.8)	26 (5.4%) (3.7–7.8)	54 (4.0%) (3.1–5.2)
Major depressive disorder	27 (3.2%) (2.2–4.6)	24 (5.0%) (3.3–7.3)	51 (3.81%) (2.9–5.0)	1 (0.2) (0.02–1.4)	25 (5.2%) (3.5–7.5)	**52 (3.9%) (3.0–5.1)**

a Any behavioural disorder (refers to oppositional defiant disorder, conduct disorder and attention deficit hyperactivity disorder)

b Any emotional disorder (generalized anxiety disorder, social anxiety disorder, separation anxiety disorder and major depressive disorder)
